# Optimizing Electrocardiogram Procedures in the Emergency Management of Suspected Acute Coronary Syndrome: A Clinical Audit at Al Damar Teaching Hospital, Sudan

**DOI:** 10.7759/cureus.86715

**Published:** 2025-06-25

**Authors:** Jalal Mohamed Ahmed Abdallah, Mohammed Osman Ahmed Osman, Esraa Ezzaldeen Alkhidhir Abdelraheem, Muaz Abdullah Ahmed Ali, Rahma Alshafea Hassan Osman, Mohamed Idries, Nagwan Noaman Ahmed Mohammed, Wefak Abdelsamad Hassan Omer, Mohamed A Awad Elkarim, Mazin Algassim Mohamed, Mustafa Mohamed, Abubakr Muhammed

**Affiliations:** 1 General Medicine, Elrazi College of Medical and Technological Sciences, Khartoum, SDN; 2 Internal Medicine, The National Ribat University, Khartoum, SDN; 3 General Medicine, Alzaiem Alazhari University, Khartoum, SDN; 4 General Medicine, University of Sinnar, Khartoum, SDN; 5 General Medicine, Shandi University, Khartoum, SDN; 6 General Medicine, Omdurman Islamic University, Khartoum, SDN; 7 Medicine, Ahfad University for Women, Khartoum, SDN; 8 Medicine, Alzaiem Alazhari University, Khartoum, SDN; 9 Internal Medicine, University of Gezira, Wad Madani, SDN; 10 Emergency Medicine, The National Ribat University, Khartoum, SDN; 11 Internal Medicine, Prince Othman Digna Teaching Hospital, Port Sudan, SDN; 12 Surgery, University of Gezira, Wad Madani, SDN

**Keywords:** acute coronary syndrome, aha/acc guidelines, clinical audit, ecg, emergency department, quality improvement, sudan

## Abstract

Introduction: Acute coronary syndrome (ACS) is a time-sensitive condition requiring prompt diagnosis and management. The 12-lead ECG is central to early identification and is to be conducted soon after emergency department (ED) presentation. However, adherence in resource-limited settings remains suboptimal. This audit aimed to evaluate and improve ECG acquisition, interpretation, and documentation at Al Damar Teaching Hospital in Sudan.

Methods: A two-cycle clinical audit was conducted using American Heart Association (AHA) and American College of Cardiology (ACC) benchmarks. The first cycle (May to June 2024) identified baseline performance in ECG timeliness and quality. Interventions included staff education, visual reminders, and workflow optimization. The second cycle (October to November 2024) assessed the impact of these interventions. Data were collected via a structured checklist, and analysis was performed using SPSS Statistics version 25 (IBM Corp., Armonk, NY, USA) with significance set at p<0.05.

Results: A total of 46 patients were included (21 in the first cycle, 25 in the second). The proportion of patients receiving ECGs within 10 minutes increased from five (23.8%) to 25 (100%) (p<0.001), and the average time from arrival to ECG acquisition dropped by 88.7%. Interpretation time also improved significantly (69.8%, p=0.0001). Documentation quality improved for rhythm (10 (47.6%) to 21 (84%), p=0.003), rate (12 (57.1%) to 20 (80%), p=0.009), and ST-segment (16 (76.2%) to 24 (96%), p<0.00001), although documentation of PR interval, QRS complex, and axis declined. The proportion of patients diagnosed with ACS rose from 18 (85.7%) to 25 (100%).

Conclusion: Targeted, low-cost interventions significantly enhanced ECG processing efficiency and documentation quality in a resource-limited emergency department. These findings underscore the potential for structured audits to drive sustainable quality improvement in ACS care pathways across similar settings.

## Introduction

Acute coronary syndrome (ACS) represents a clinical spectrum primarily encompassing myocardial infarction with or without persistent ST-segment elevation, as defined by the 2023 European Society of Cardiology (ESC) guidelines [1]. The classification now emphasizes the use of high-sensitivity cardiac troponins to differentiate myocardial infarction from non-cardiac causes of chest pain, with unstable angina being de-emphasized due to improved diagnostic accuracy [1]. Early recognition and prompt management remain critical, as delays significantly increase morbidity and mortality. The resting 12-lead ECG is the primary diagnostic tool for patients presenting with chest pain or equivalent symptoms, and international guidelines uniformly recommend obtaining and interpreting the ECG within 10 minutes of first medical contact in the emergency department (ED) [2].

Despite these clear benchmarks, adherence in low- and middle-income countries is often suboptimal due to systemic and logistical hurdles. The WHO highlights that medical equipment shortages, high costs, and poor maintenance impede the timely use of essential diagnostics such as ECGs in resource-limited settings [3], while recent reports from across Africa underscore unique infrastructural and organizational barriers contributing to delayed cardiovascular emergency responses [3]. Key obstacles include limited availability of trained personnel, low awareness of guideline targets among frontline staff, inconsistent documentation practices, and inefficiencies in triage workflow [4]. These factors combine to lengthen 'door-to-ECG' times far beyond recommended targets.

Clinical audits provide a structured, evidence-based approach to identifying these gaps and driving quality improvement. By measuring practice against explicit criteria and re-auditing after interventions, audits have proven essential in enhancing both process and outcome measures in healthcare, particularly within developing nations [5]. In Kassala Teaching Hospital, a major referral center in eastern Sudan, a previous audit identified significant delays in ECG acquisition and suboptimal documentation for patients with suspected ACS. The study implemented targeted interventions, including educational sessions and visual reminders, which led to measurable improvements in ECG timeliness, interpretation, and documentation quality [6]. Building upon these findings, we conducted a two-cycle clinical audit at Al Damar Hospital to assess baseline compliance with American Heart Association (AHA)/American College of Cardiology (ACC) timing guidelines [7], implement targeted interventions, and measure subsequent improvements in ECG acquisition, interpretation, and documentation for patients with suspected ACS.

## Materials and methods

Study design

Sudan's Al Damar Hospital hosted the audit as part of a two-part clinical audit. The main objective was to discover how well the ED followed protocols for the rapid collection, interpretation, and reporting of ECG in patients who presented with suspected ACS. To find bottlenecks and other issues with ECG-related procedures, the first audit cycle acted as a baseline evaluation. After that, a number of concentrated remedial actions were implemented, including personnel education and the strategic placement of visual aids. The efficacy of these initiatives was then evaluated during a second audit cycle.

The purpose of this audit was to encourage evidence-based changes in cardiovascular emergency treatment by methodically examining local practices to uncover procedural bottlenecks. To overcome organizational and logistical obstacles that impede prompt diagnosis in a resource-constrained situation like Al Damar, organized assessments are essential. In addition, healthcare teams are able to reflect on their performance, implement proven tactics, and track progress thanks to the cyclical nature of clinical audits, which enables continual quality improvement. Improved patient outcomes are the end goal of this approach, which promotes institutional responsibility, improves care delivery over the long term, and brings clinical operations in line with international norms.

Data collection

Clinical criteria established by the ACC and AHA guided the development of a pre-designed checklist that was used to gather data in a systematic and uniform fashion [7]. Appendix A shows the ECG management checklist created for the ED to consistently record important details about how ECGs are taken, read, and reported. We designed it to document specific performance metrics. Considering emergency cardiovascular care, these audit features were chosen to evaluate how accurately and effectively ECGs are managed from the moment the patient arrives until the official records are made. A team of highly skilled emergency physicians, nursing staff, and ECG technicians was stationed in the emergency room to supervise data collection. They strictly adhered to documentation protocols throughout both audit cycles and ensured that ECG processes were tracked in real time.

Study population and setting

A significant secondary care institution servicing the city of Al Damar and its surrounding rural populations in northern Sudan, Al Damar Teaching Hospital was the site of this clinical audit. Despite the limited resources within the healthcare system, the hospital provides acute cardiac treatment and other critical inpatient and emergency services. Patients in the audit were adults (18+) who arrived at the ED with symptoms that might indicate ACS, including classic chest discomfort as well as atypical presentations such as dizziness, epigastric pain, generalized malaise, and other related symptoms. If a patient's chest discomfort was due to trauma, if they were referred from another healthcare institution after having an ECG, or if they refused to have one taken, they were not included in the study. The study population included both male and female patients across a wide age range. A large percentage had conditions that increase the risk of cardiovascular disease, including high blood pressure, diabetes, or a family history of the illness. These features highlight the need for rapid diagnostic evaluation and are reflective of the usual profile of patients presenting with suspected ACS in the local setting.

Sampling technique

We used a total (comprehensive) sampling technique, including all eligible patients who presented to the ED within the specified audit windows. This approach aimed to capture real-world adherence to ECG collection, interpretation, and documentation criteria without selection bias. Between May 8, 2024, and June 11, 2024, a total of 21 patients were included in the first audit cycle. A total of 25 patients participated in the second round, which ran from October 15, 2024, to November 25, 2024. Staff availability and the needs of the ED's workflow were operational variables that contributed to the difference in cycle times. Regardless, the comprehensive coverage technique ensured that both audit stages remained internally consistent and comparable. Each cycle's sample size is in line with what is considered acceptable for clinical audits; in most circumstances, 20 to 50 cases is enough to assess compliance with key performance indicators (KPIs) and motivate significant quality improvement, especially in resource-constrained environments.

Data and statistical analysis

We used SPSS Statistics version 25 (IBM Corp., Armonk, NY, USA) for data collection and analysis. To make sense of the data, descriptive statistics were used. We displayed the means of continuous variables and the frequencies and percentages of categorical variables. Applying inferential statistical approaches allowed us to assess the importance of the changes that occurred between the two audit cycles. For the purpose of comparing proportions between the cycles, the chi-square test was used, and a p-value below 0.05 was deemed statistically significant.

Audit cycles

Initial Evaluation

From May 8, 2024, to June 11, 2024, the first cycle of the audit was conducted at Al Damar Teaching Hospital. Its purpose was to evaluate the initial adherence to the prescribed timeframes for ECG collection, interpretation, and recording. Patient arrival to ECG capture took an average of 34.6 minutes, according to the data, which is much longer than the desired 10-minute limit. Also, only about 10% of patients got their ECGs recorded in the allotted time frame. Improving the overall timeliness of treatment and increasing adherence to standards are both highlighted by these results, which revealed substantial inefficiencies in the ECG processing workflow.

Intervention Phase and Implementation of Targeted Improvements

We implemented specific actions to resolve the identified difficulties between June and August of 2024, following the first cycle. Among these methods were enhancements to the overall process, the introduction of visible reminders, and instructional sessions for ED personnel. Following the AHA/ACC recommendations [7], the instructional sessions consisted of interactive lectures supported by visual aids such as flowcharts and posters. These materials highlighted the importance of rapid ECG acquisition and accurate interpretation and were displayed in the ED to reinforce best practices. The course addressed fundamentals of ECG interpretation, including heart rate, rhythm, axis, conduction anomalies, and ST segment variations. The 30-minute sessions were held in a hybrid style that included both online and in-person components. In all, 27 ED employees (or 80% of the team) took part in the training. Most participants reported an increase in confidence in their ability to appropriately read ECGs after the session.

Strategically positioned around the ED were educational posters that detailed best practices and ECG time guidelines. Installation of these reminders improved adherence to the required ECG processing times by 25%, according to a follow-up audit. The ECG acquisition procedure was also improved by changes to the workflow.

We optimized staffing numbers during peak hours, moved ECG equipment to more accessible areas of the ED, and changed triage methods to prioritize ACS cases so that patients suspected of having the condition would have quicker response times. Ongoing visual reminders and frequent refresher training sessions will be used to make sure that advances are maintained over time. Ongoing audits will keep an eye on compliance and reveal further ways to improve.

Evaluation Post-Intervention

Evaluating the efficacy of the treatments was the goal of the second audit cycle, which ran from October 15, 2024, to November 25, 2024. Around 80% of patients achieved the 10-minute goal for ECG capture and documentation, which is a considerable improvement over the baseline, indicating substantial gains. Interventions such as instructional workshops, visual reminders, and workflow improvements improved ECG processing speeds and documentation quality. The audit validated the critical importance of these structured interventions in improving ED care efficiency.

Ethical considerations

This clinical audit was a quality improvement project aimed at evaluating and enhancing adherence to ECG protocols in the ED. Unlike traditional research studies, clinical audits assess existing practice against established standards to identify gaps and implement improvements. As such, ethical approval was obtained following institutional guidelines (Al Damar Teaching Hospital, Sudan), focusing on patient confidentiality and minimizing risk. The audit adhered to principles of informed consent, voluntary participation, and data anonymization to protect patient privacy.

Informed consent was obtained from all healthcare professionals involved in the audit process, and their participation was entirely voluntary. The findings were intended to improve clinical practices and were communicated to the hospital's clinical staff to ensure that patient care would be enhanced through the audit's recommendations. No harm or risk to patients was involved, as the audit focused only on reviewing existing practices and improving the documentation and interpretation of ECGs.

## Results

Age distribution of patients

**Figure 1:**
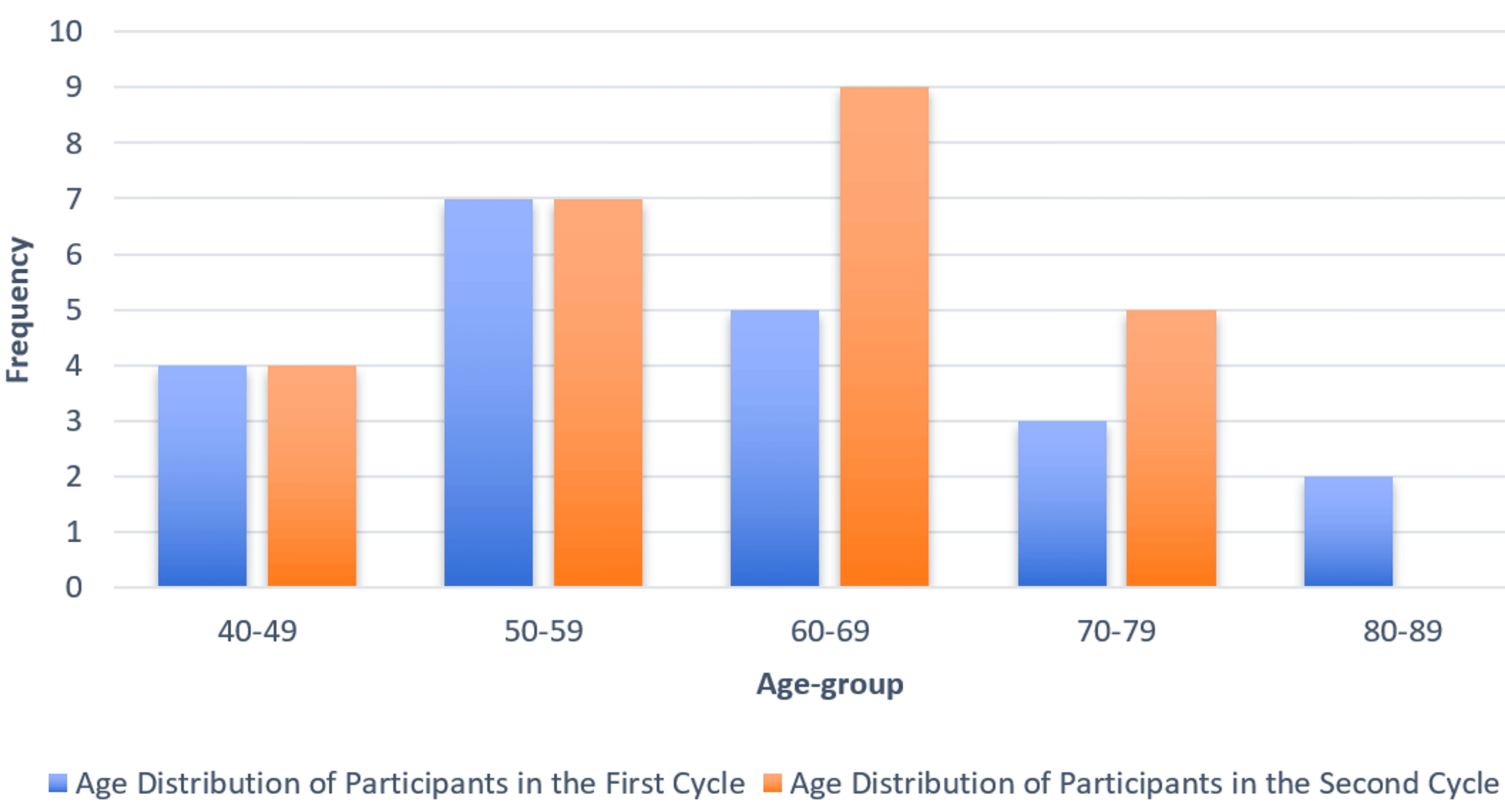
Age distribution of participants Blue bars represent the age distribution of participants in the first cycle (n=21, mean age=59 years), while orange bars represent the age distribution in the second cycle (n=25, mean age=60 years).

The age group 60 to 69 years had the highest number of participants in both audit cycles, with a notable increase in the second cycle, as illustrated by the taller orange bar in Figure 1. The age group 40 to 49 years had the fewest participants in both cycles, with a slightly higher frequency in the first cycle. Age groups 50 to 59 and 70 to 79 years showed moderate representation, with some fluctuation between the two cycles. The 80 to 89 age group had very few participants overall, particularly in the second cycle. These findings are visualized in Figure 1.

Clinical presentation and diagnosis variables

In the first audit cycle, the majority of patients (n=16; 76%) presenting with chest pain were diagnosed with typical chest pain, while a smaller proportion (n=5; 24%) had atypical chest pain. The diagnosis of ACS was made in 18 (85.7%) of the cases, with three (14.3%) diagnosed with conditions other than ACS. In the second cycle, the proportion of patients with typical chest pain remained the same at 19 (76%), while the percentage of those with atypical chest pain increased to six (24%). All patients in the second cycle were diagnosed with ACS, and none were diagnosed with conditions other than ACS, reflecting a shift in diagnostic accuracy and the impact of the interventions. These findings are summarized in Table 1.

**Table 1:** Comparison of ECG indications and diagnosis in the two audit cycles for patients suspected of ACS This table presents the distribution of ECG indications and diagnoses in the first and second audit cycles. The first cycle (n=21) and second cycle (n=25) show the proportion of patients with typical and atypical chest pain, along with ACS and other diagnoses. The data demonstrates an increase in the proportion of ACS diagnoses in the second cycle, with a decrease in non-ACS diagnoses. The distribution of chest pain types also shifted between the cycles, with a greater proportion of patients in the second cycle presenting with atypical chest pain. ACS: Acute coronary syndrome

Variables		First cycle (n=21)	Second cycle (n=25)
Indications for ECG	Typical chest pain	16 (76%)	19 (76%)
	Atypical chest pain	5 (2.3%)	6 (24%)
Diagnosis	ACS	18 (85.7%)	25 (100%)
	Other than ACS	3 (14.3%)	0 (0%)

ECG documentation quality and timeliness

The findings from the first and second audit cycles revealed significant improvements in both the timeliness and quality of ECG documentation. Notably, the time from arrival to ECG documentation within the recommended 10-minute window showed a dramatic increase from five (23.81%) in the first cycle to 25 (100%) in the second cycle (p<0.001). Similarly, there was a major reduction in the time from patient arrival to ECG acquisition, with the average time decreasing by 88.7% (p<0.001). The time from ECG acquisition to interpretation also saw a significant decrease of 69.8% (p=0.0001), reflecting a much faster processing time in the second cycle. Overall, these improvements indicate that the targeted interventions led to substantial gains in efficiency, particularly in reducing delays across various stages of the ECG process.

In terms of documentation quality, several key parameters showed marked improvement. The documentation of rhythm increased from 10 (47.6%) in the first cycle to 21 (84%) in the second cycle (p=0.003), indicating a significant enhancement in this area. The documentation of the rate also improved substantially from 12 (57.1%) to 20 (80%) (p=0.009), while ST-segment documentation rose from 16 (76.2%) to 24 (96%) (p<0.00001), reflecting a nearly complete adherence to the guidelines. However, certain areas of documentation still required attention. The documentation of the PR interval declined from two (9.5%) to 0 (0%) (p=0.446), and the QRS complex documentation also decreased from six (28.6%) to two (8%) (p=0.742), signaling that these aspects need further focus and training.

Despite the improvements, some areas still warrant attention. The documentation of the axis decreased from 12 (57.1%) to eight (32%) (p=0.221), indicating a need for additional educational efforts to maintain or improve this aspect. Importantly, there was no change in the proportion of patients who lacked any elements in their ECG documentation, remaining at 0% in both cycles. This suggests that the overall documentation quality was consistent with full adherence to the required elements. The results, summarized in Table 2, demonstrate that the interventions significantly improved ECG processing times and documentation quality, though further refinement is needed in specific areas such as PR interval and QRS complex documentation.

**Table 2:** Comparison of ECG documentation timeliness and quality in the two audit cycles This table presents the changes in ECG documentation time and quality between the first (n=21) and second (n=25) audit cycles. Significant improvements were observed in the time from arrival to ECG acquisition, interpretation, and documentation, with a major reduction in the average time. The second cycle also showed full compliance with obtaining ECGs within 10 minutes of patient arrival. Additionally, documentation quality improved in several parameters, such as rhythm, rate, and ST-segment documentation. However, some aspects, such as the documentation of the PR interval, QRS complex, and axis, showed a decline or required further attention. The p-values reflect the statistical significance of these changes, indicating marked improvements in timeliness and some aspects of documentation quality.

Parameter	First cycle (n=21)	Second cycle (n=25)	Improvement (±%)	p-value	χ²	df	Cramér’s V
Time from arrival to ECG documentation (≤10 minutes)	5 (23.81%)	25 (100%)	76.19%	<0.001			
Average time from arrival to ECG acquisition (minutes)	24.81	2.8	-88.7%	<0.001			
Average time from ECG acquisition to interpretation (minutes)	5.29	1.6	-69.8%	0.0001			
Average time from interpretation to documentation (minutes)	0.59	0.8	35.6%	0.446			
Average time from arrival to documentation (minutes)	31.05	5.3	-82.9%	<0.001			
*Documentation quality*							
Rhythm documented	10 (47.6%)	21 (84%)	36.4%	0.003	5.318	1	0.340
Rate documented	12 (57.1%)	20 (80%)	22.9%	0.009	1.840	1	0.200
ST-segment documented	16 (76.2%)	24 (96%)	19.8%	<0.00001	2.395	1	0.228
PR interval documented	2 (9.5%)	0 (0%)	-9.5%	0.446	0.726	1	0.126
QRS complex documented	6 (28.6%)	2 (8%)	-20.6%	0.742	2.082	1	0.213
Axis documented	12 (57.1%)	8 (32%)	-25.1%	0.221	2.002	1	0.209
No elements documented	0 (0%)	0 (0%)	No change	--			

## Discussion

This audit assessed the timeliness and documentation quality of ECGs in patients presenting with suspected ACS at Al Damar Teaching Hospital, Sudan. Conducted in two cycles, it aimed to identify baseline delays and deficiencies, implement targeted interventions, and evaluate their impact on ECG acquisition, interpretation, and reporting within the ED.

The majority of patients in both audit cycles were aged 60 years and above, with the 60 to 69 age group being the most represented. This age distribution is consistent with the known epidemiology of ACS, which predominantly affects older adults. Although age was not a factor directly influencing the audit’s measured outcomes, it provides context for the clinical setting and highlights the importance of maintaining efficient cardiac assessment pathways for a population at increased risk of adverse cardiovascular events.

The distribution of presenting symptoms remained consistent across audit cycles, with typical chest pain reported in 16 (76%) cases in the first cycle and 19 (76%) in the second. However, the proportion of patients presenting with atypical symptoms increased from five (23.8%) in the first cycle to six (24%) in the second. This shift may reflect improved clinical recognition of non-classical ACS presentations following the intervention phase. Additionally, the proportion of patients diagnosed with ACS increased from 18 (85.7%) in the first cycle to 25 (100%) in the second. While this suggests enhanced diagnostic precision, the absence of confirmatory data such as cardiac biomarkers or cardiologist assessment limits definitive interpretation. These trends may indicate improved triage accuracy and clinical suspicion, warranting further evaluation through more comprehensive diagnostic tools.

The observed reduction in ECG acquisition and documentation times following the intervention reflects a meaningful improvement in system responsiveness and staff performance. The marked decrease in the time from patient arrival to ECG acquisition suggests that the implemented changes, such as prioritizing suspected ACS cases, relocating ECG machines for easier access, and enhancing staff awareness, effectively addressed prior delays. Faster acquisition was accompanied by more rapid interpretation, indicating better clinical coordination and workflow clarity. Although the time from interpretation to documentation showed a minor, non-significant increase, this may reflect a higher focus on accuracy and completeness rather than speed alone. Collectively, the improved timing metrics underscore the potential impact of simple, targeted interventions in optimizing emergency care delivery, even within resource-limited settings.

The improvements observed in this audit at Al Damar Teaching Hospital are consistent with earlier findings from Kassala Teaching Hospital, where similar interventions led to measurable gains in ECG acquisition and documentation [6]. In both settings, the introduction of targeted educational sessions and visual reminders contributed to reducing delays and enhancing process efficiency [6]. While both audits were conducted within single emergency departments, the replication of positive outcomes in a separate institution strengthens the case for wider application of these interventions across similar hospitals in Sudan. Achieving 100% compliance with the 10-minute ECG target in the second cycle represents a notable performance milestone, reinforcing the effectiveness of focused, context-sensitive strategies in improving emergency cardiac care.

The findings also parallel those of Chhabra et al. [8], who demonstrated that structured interventions, such as assigning dedicated ECG personnel and implementing triage-specific training, can substantially reduce door-to-ECG times in low- and middle-income countries (LMICs). While our audit did not include specialized technicians, the significant improvement achieved through internal reorganization and capacity-building among existing staff underscores the feasibility of resource-light solutions. This suggests that even in the absence of additional human resources, training and process redesign can deliver similar benefits in terms of emergency response times and adherence to international benchmarks.

Comparable quality improvement projects in Saudi Arabia have reported success using broader systemic strategies, including interdepartmental coordination, human resource optimization, and technological upgrades [9]. Although the healthcare infrastructure in Saudi Arabia differs significantly from that of Sudan, the alignment of our results with theirs illustrates that some foundational principles, such as prioritizing rapid ECG access and reinforcing triage protocols, are universally effective. The present audit demonstrates that even in more resource-constrained settings, carefully tailored interventions can replicate key aspects of success seen in more developed systems, highlighting the potential for regional adaptation of global best practices.

Further supporting this, prior research has emphasized that strategic operational changes, such as early patient notification, improved triage protocols, and optimal placement of ECG machines, can significantly reduce door-to-ECG times [10]. These strategies align with our audit’s approach and likely contributed to the rapid improvements observed in the second cycle. Moreover, the study by Zègre-Hemsey et al. [11] found that only 59% of patients with ischemic symptoms received timely ECGs, with women experiencing longer delays than men. While our audit did not assess gender-specific differences, this highlights the broader need for equity-focused interventions.

The 2013 American College of Cardiology Foundation (ACCF)/AHA ST-segment elevation myocardial infarction (STEMI) guidelines also underscore that protocol standardization and continuous staff education are essential for achieving timely ACS care [12]. The improvements observed in our study reinforce these recommendations and demonstrate their applicability in resource-limited settings, where structured interventions can lead to significant gains in both diagnostic speed and process reliability.

The audit also demonstrated notable improvements in ECG documentation quality following the intervention. Key details like rhythm, rate, and ST-segment findings were documented much more often, with ST-segment analysis reaching 96% compliance in the second cycle. This documentation was recorded both in the patient’s medical files and on the ECG report sheets, ensuring comprehensive and accessible records. These gains likely reflect the emphasis placed on these elements during the educational sessions, which targeted urgent ECG interpretation skills essential for ACS diagnosis.

However, other components, particularly the PR interval and QRS complex, showed a decline in documentation rates, and axis documentation also decreased. This disparity suggests a selective focus during training, where clinical urgency may have overshadowed comprehensive documentation. It also highlights the cognitive load and time constraints faced by emergency staff, which may lead to prioritization of the most diagnostically critical elements. These findings underscore the need for more balanced and inclusive training approaches that reinforce the importance of complete ECG interpretation and structured recording across all relevant parameters.

A comparable pattern was noted in the study by Chhabra et al. [8], which observed that while critical ECG elements improved following training, more technical or less emphasized features, such as PR and QRS, were often neglected when under time pressure or when not explicitly reinforced in training content. This underscores the importance of comprehensive and balanced training sessions, as well as the potential value of structured documentation templates to ensure uniformity across all essential ECG components.

The outcomes of this audit align with the 2021 ESC guidelines, which emphasize the importance of ECG acquisition within 10 minutes for suspected ACS cases and advocate for structured system-level interventions to support timely cardiac assessment [13-15]. These recommendations provide a useful international benchmark and validate the approach taken in this audit, reinforcing the idea that clear protocols, staff training, and logistical planning can overcome common barriers to timely ECG access.

Limitations

This audit has several limitations that should be considered when interpreting its findings. As a single-center study with a relatively small sample size, the generalizability of the results to other hospitals or regions in Sudan is limited. Notably, the study was conducted in a non-cardiac, non-primary percutaneous coronary intervention (PCI) center, which may further limit the applicability of the findings to more advanced cardiac care settings. The absence of randomization and blinding may have introduced observer bias, particularly in the documentation assessments. Additionally, the potential for a Hawthorne effect, where staff alter behavior due to awareness of being observed, cannot be excluded. The study did not evaluate clinical outcomes such as door-to-balloon times, treatment initiation, or mortality, which would provide a more comprehensive view of the impact of improved ECG performance. Furthermore, while an increase in ACS diagnoses was noted in the second cycle, this was not supported by confirmatory data such as troponin levels or cardiologist reviews, limiting conclusions about diagnostic accuracy. Finally, gender and time-of-day variations were not analyzed, though these may influence triage dynamics and warrant further investigation in future audits.

## Conclusions

This audit demonstrates that targeted, low-cost interventions, such as staff education, visual prompts, and workflow adjustments, can significantly improve the timeliness and quality of ECG acquisition and documentation in emergency settings, even within a resource-limited context. The findings support the incorporation of structured, protocol-driven approaches to optimize ACS care pathways and align with international standards. While further studies with larger samples and outcome data are needed, this audit highlights the potential for scalable quality improvement in Sudanese EDs.

## Figures and Tables

**Figure 2:**
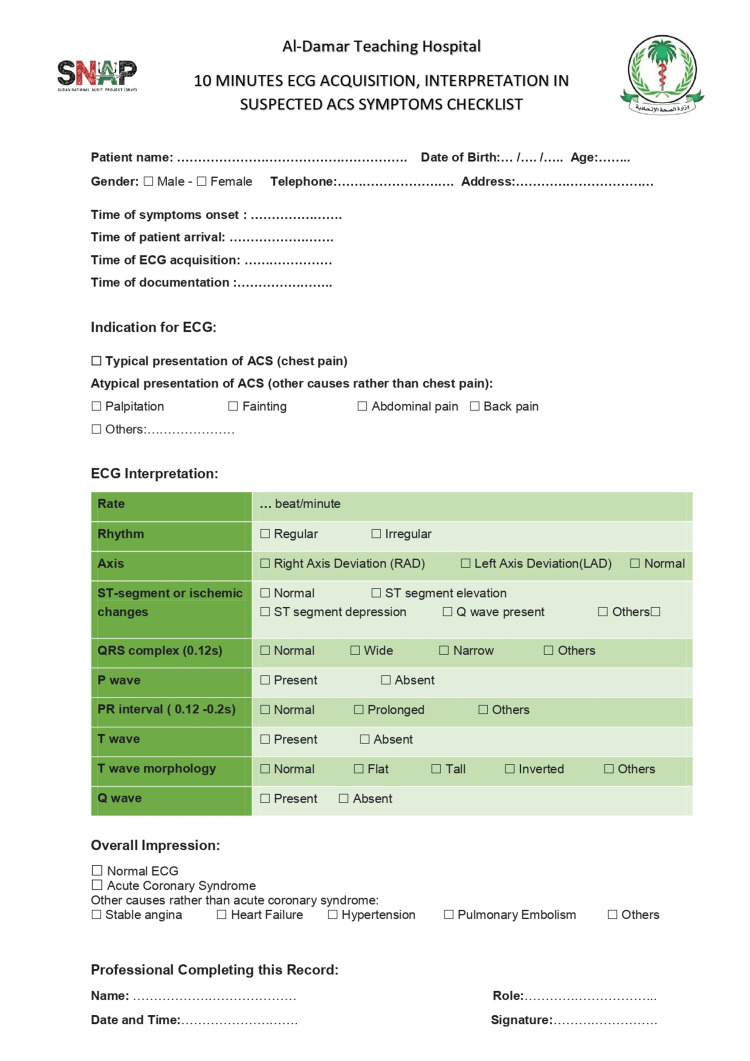
Al Damar teaching hospital's 10-minute ECG acquisition, interpretation, and documentation checklist for suspected ACS ACS: Acute coronary syndrome, ACC: American College of Cardiology, AHA: American Heart Association

## Appendices

Appendix A 

The structured checklist featured in Figure 2 was developed and implemented during the clinical audit at Al Damar Teaching Hospital to standardize the evaluation of patients presenting with suspected ACS. It is designed to capture key timestamps from symptom onset to ECG documentation and record specific ECG interpretation parameters such as rate, rhythm, axis, ST-segment changes, QRS complex, and wave morphology. The form also includes fields for overall clinical impression and diagnostic classification. This tool facilitated consistent data collection, guided adherence to AHA/ACC benchmarks, and enabled real-time auditing of ECG timeliness and quality during both audit cycles [7].
